# Determinants of community-based health insurance implementation in west Gojjam zone, Northwest Ethiopia: a community based cross sectional study design

**DOI:** 10.1186/s12913-019-4363-z

**Published:** 2019-08-02

**Authors:** Tsega Hagos Mirach, Getu Debalkie Demissie, Gashaw Andargie Biks

**Affiliations:** 1 Department of Health Systems and Policy, College of Medicine and Health Sciences, University of Gondar, Gondar, Ethiopia; 2 Department of Health Education and Behavioral Sciences, College of Medicine and Health Sciences, University of Gondar, Gondar, Ethiopia

**Keywords:** Community based health insurance, Enrollment, Determinants, West Gojjam

## Abstract

**Background:**

In most developing countries, healthcare cost is mainly paid at the time of sickness and out-of-pocket at the point of service delivery which potentially could inhibit access**.** The total economic cost of illness for households is also estimated to be frequently above 10% of household income which is categorized as catastrophic. The purpose of this study was to assess factors that determine decisions to join the community based health insurance in West Gojjam zone.

**Methods:**

A community based cross sectional survey was conducted to collect data from 690 household heads using a multistage sampling technique. A binary logistic regression was used to identify the determinants of household decisions for CBHI enrollment.

**Results:**

Out of the participants, 58% were CBHI members. Besides, family size (AOR = 1.17; CI = 1.02–1.35), average health status (AOR = .380; CI = .179–.805), chronic disease (AOR = 3.42; CI = 1.89–6.19); scheme benefit package adequacy (AOR = 2.17; CI = 1.20–3.93), perceived health service quality (AOR = 3.69; CI = 1.77–7.69), CBHI awareness (AOR = 4.90; CI = 1.65–14.4); community solidarity (AOR = 3.77; CI = 2.05–6.92) and wealth (AOR = 3.62; CI = 1.67–7.83) were significant determinant factors for enrolment in the community based health insurance scheme.

**Conclusion:**

CBHI awareness, family health status, community solidarity, quality of service of health institutions, and wealth were major factors that most determine the household decisions to enroll in the system. Therefore, in-depth and sustainable awareness creation programs on the scheme; stratified premium- based on economic status of households; incorporation of social capital factors, particularly building community solidarity in the scheme implementation are vital to enhance sustainable enrollment. As perceived family health status and the existence of chronic disease were also found significant determinants of enrollment, the Government might have to look for options to make the scheme mandatory.

## Background

In most developing countries, healthcare cost is mainly paid at the time of sickness and out-of-pocket (OOP) at the point of service delivery which potentially could inhibit access [1]. The share of health expenditure to households’ income is also considered significant. The total economic cost of illness for households is estimated to be frequently above 10% of household income. This is potentially catastrophic: as such expenditure levels are “likely to force households to cut their consumption of other minimum needs, trigger productive asset sales or high levels of debt, and lead to impoverishment” [2].

Rural households in most developing countries are excluded from the formal insurance system. Thus, community-Based Health Insurance (CBHI) is being promoted for its potential to pool risks and resources so as to reduce households out off pocket expenditure and improve access to health care [3].

The scheme is being implemented in most low and middle income countries. However, it is continued to be challenged by low uptake, coverage, and sustainability issues. As the findings showed, a different interconnected factors at different levels (individual, institutional and policy) determine the application of CBHI schemes [4].

The Ethiopian health care system is characterized by High out-of-pocket expenditure, increased health care needs, inability to mobilize more resources for health among rural dwellers, and inability to fully recover costs of care incurred by beneficiaries [5]. Thus, the country introduced a pilot community based health insurance (CBHI) scheme in 2011. CBHI uptake was so remarkable in the country in the piloting year which was raised to 41% in one year. However, in the next year 18% of the households which enrolled in the first year withdrawing their payment [6].

Enrollment determinant factors are broadly classified as household charactertics, scheme related factors, social capital, institutional factors, and supply side factors [7]. Most studies reported that the role of education was statistically significant in determining decisions to enroll [8–10]. Studies in Tanzania and Ethiopia, on the otherhand, noted that income had a positive significant contribution to making decisions about enrolment [9, 11].

Raising awareness and understanding the risk pooling principle (the process of creating a common pool of money so that the financial risks entailed by certain high-risk individuals are mitigated by money from low-risk individuals) is another vital issue to enhance enrollment. Studies conducted in different African countries such as Tanzania and Ethiopia found failure to understand the risk pooling principle and lack of information limit enrolment [6, 11]. Besides, having a favorable policy of environment that provideds appropriate legislative frameworks, integrating CBHI schemes with government structures, and sustained technical assistance are among the vital factors for a successful implementation of the scheme [12].

In CBHI scheme implementation, lack of a systematic incorporation of the social context is justified for low rates of enrolment [13]. Different studies revealed that there must be social capital in the community for CBHI to have a long-term effect [14–16]. Scheme related factors, such as the benefit package design, premium amount, and transparency affects people’s decision to enroll [17].

Despite the significance of customized designs and evidence based implementation of the scheme, only limited studies focus mainly on willingness to pay, enrolment, and dropout rates were done in Ethopia. Therefore, the aim of this study was to identify the determinants of household CBHI enrollment.

### Hypothesis of the study

According to a priori theoretical ground, factors such as larger family size, poor perceived health status, good insurance awarness, adequacy of benefits provided by the scheme, quality of health service delivery, existence of good scheme regulatory and complaint handling mechanisms, good social capital, and high household wealth were assumed to increase household’s probability of deciding to enroll.

## Methods

### Study design and setting

A Community based cross sectional study design was used. The study site is located in West Gojjam zone in Amhara region 385 km from Addis Ababa, northwest Ethiopia. The total population of the zone is estimated at 2,474,254 (1,220,477 male and 1,253,777 female) [18].

There are 13 rural districts and 5 town administrations in West Gojjam zone. Regarding to health service institutions; there are one government hospital, 90 health centers, 76 private health facilities (hospitals and higher clinics), and 363 health posts [19]. A community based cross-sectional study was conducted particularly in rural kebeles (the smallest administrative unit in Ethiopia) in March 2017.

### Study participants and data collection

Household heads who were residents (lived for more than 6 months in the kebelles) of the selected districts were included; heads and/or spouses employed in the formal sectors were excluded.

The sample size was determined using the single population proportion formula by assuming a 58% proportion (based on the the 2015 Ethiopian Insurance Agency pilot study, which reported that 58% of the households in the study area were members of the scheme) [20], a 5% margin of error, a 95% confidence level, and 5% non-response rate.

$$ n=\frac{Z_{a/2}^2\ast P\ast \left(1-P\right)}{E^2} $$

A total of 690 household heads participated in the study. A multistage sampling procedure was used to select participants. Firstly, two of the 10 CBHI implementing districts were randomly selected. Secondly, six kebelles were randomly selected out of the two districts. Thirdly, household heads were selected by the systematic random sampling technique from each kebelle. Finally, the lottery method was used to select a number between one and the sampling interval which turned out to be 29.

A cross sectional survey was carried out by using a semi-structured questionnaire to gather data from selected representative households in the zone. The primary data was collected via an enumerator-administered questions which comprised, among others things, household characteristics, scheme related factors, the benefit package design, social capital, institutional and supply side factors which are considered to be important variables affecting household CBHI enrollment.

### Data quality and analysis

The instrument was drafted in English and translated to Amharic and back to English by language experts.

Besides, to maintain its consistency, the questionnaire was pre- tested on local people living outside the selected kebelles. and cronbach’s alpha average value of variables was done.

Epi-info version 3.5 was used for data entry. Descriptive statistics, such as frequency distributions, tables, and graphs were used to present the characteristics of the data. Bi-variable analysis was performed to assess the strength of association of the independent variables with the dependent variable, and all the variables were fitted for the multivariate analysis. The effects of the several independent variables on CBHI enrollment decision were assessed through the multivariable analysis, and STATA 10 statistical package was used for the logistic regression model.

To estimate the strength of association between the independent variables and the CBHI enrollment odds ratio, a 95% C. I, and *p*-values were used.

Besides, different diagnostic tests were done, particularly goodness of fit of the model by the Hosmer and Lemeshow test; (where p-value of 0.0822 was found), Variance inflation factor (VIF) for the multicollinearity test (the mean VIF result was 1.39), and sample size sufficiency test (which found 15 sample size for each case by dividing the 690 sample by the total number of cases which in this case was 46) was also done to increase estimator accuracy and control confounding effects as logistic regression can control numereous confounders for large sample size.

The number of people included in the multiple logistic regression was 648 with a missing value of 6.3% which was treated through the imputation technique (using mean value for a continuous variables and mode for categorical variables).

### Operational definitions and measurement

The scale and measurement for most of the variables used in this study were adapted from the Federal Health Insurance Agency of Ethiopia pilot study 2015 [20]. However, variables such as wealth and social capital were taken from EDHS 2011 and World Bank group respectively.

#### CBHI awareness

This referes to household heads knowledge of CBHI existence, its principles, and significance. It was assessed by asking the participants five sets of related questions. The Cronbach’s alpha value was .722.

#### Benefit-package design

Refers to the sufficiency of the health services that are offered by the scheme that would be covered through out-of-pocket spending at times of sickness; and the premium amount charged. This was measured by two questions using the Likert scale with a Cronbach’s alpha value of .673.

#### Quality of service delivery

This includes health workers` attitude, availability of drugs, facilities/ medical equipment, waiting time, and the rapidity of the treatment’s results of the health service provider; and measured via eleven Likert scale type questions. The cronbach’s alpha result of this variable was .73.

#### Scheme related factors

Refer to transparency on scheme rules, regulation, and procedures measured through one Likert scale type question with a Cronbach’s alpha value of .784.

#### Institutional factors

Include *r*egulatory mechanisms, complaint handling systems, and insurance education. This variable was measured by seven Likert scale type questions. The Cronbach’s alpha result of this variable was .688.

#### Supply side factors

Refer to the quality of care and distance of household’s homes from the nearest health facility which is measured by eleven Likert scale type questions with a Cronbach’s alpha value of .786..

#### Social capital

Includes trust, networks and group participation, social norms, and solidarity and togetherness features of the social organization of the community. Fifteen Likert scale type questions were adopted from the World Bank Group [21] to measure the variable with Cronbach’s alpha value of .656.

#### Wealth

Refers to household assets and was measured by questions adopted from EDHS 2011 [22]. Principal Components Analysis (PCA) in STATA was used to develop the index.

## Results

### Socio demographic characteristics of the study participants

A total of 690 household heads with 97% response rate participated in the study. Of the participants, 598 (89.7%) were male with a mean age of 45.4 (± 12.09). The majority of respondents were Orthodox Christian by religion, and married (Table 1).

**Table 1:** Demographic and Socio-economic characteristics of the study participants in West. Gojjam Zone, northwest Ethiopia, 2017 (*n* = 690)

Variable	Description	Frequency n (%)
Sex	Male	598 (89.7)
	Female	62 (10.3)
Age	Less than 30 years	46 (7.1)
	30–39 years	165 (25.7)
	40–49 years	186 (28.9)
	50–59 years	165 (25.7)
	60 years and above	80 (12.5)
Marital status	Married	561 (84.1)
	Single	26 (3.9)
	Divorced	42 (6.3)
	Widowed	24 (3.6)
Education	Not able to read and write	83 (15.5)
	Able to read and write	228 (42.7)
	Primary school [1–8]	169 (31.6)
	Secondary school & above	54 (10.1)
Occupation	Farmers	636 (95.4)
	Merchants	6 (0.9)
	Housewifes	1 (0.1)
	Daily laborers	13 (1.9)
	Others	3 (0.4)
Family size	≤ 3 members	134 (20.7)
	4–6 members	342 (53)
	> 6 members	170 (26.3)
Household’s wealth	Poorest (1st quintile)	148 (23.2)
	Poor (2nd quintile)	11 (17.4)
	Medium (3rd quintile)	129 (20.2)
	Rich (4th quintile)	127 (19.9)
	Richest (5th quintile)	124 (19.4)

The majority of the respondents were farmers, a mean family size was 5.38 (± 2.79), 42.7% were able to read and write, and  the household wealth of 23% of the respondents was in the first quintile.

### Factors associated with CBHI enrollment

The outcome variable (CBHI enrollment decision) was treated as a binary outcome: “1” for enrolled and “0” for un-enrolled households and 19 explanatory variables were considered in the econometric model. The econometrics logistic analysis showed that household family size was significantly associated with CBHI enrollment. As the household size increased by one unit, the likelihood to join the scheme increased by 1.17 times (AOR = 1.17; CI = 1.02–1.35). Likewise, participants with good CBHI awareness were 3.77 times more likely to join the scheme compared with their counterpart (AOR = 4.90; CI = 1.65–14.4).

The results also indicated that household with good perceived family health status were 0.38 times less likely to enroll compared with those with poor family health (AOR = .380; CI = .179–.805). Similarly, household who had family members with chronic disease were 3.42 times more likely to join compared with their counterparts (AOR = 3.42; CI = 1.89–6.19) (Table 2).

**Table 2:** Logistic regression analysis of willingness to join CBHI in West Gojjam Zone, 2017

Variables	Enrollment		Crude OR (95% CI)	Adjusted OR (95% CI)	Marginal effects
	Yes n (%)	No n (%)			
District					
*Jabi-tehnan*	276 (63)	162 (37)			
*Burie*	104 (48.3)	111 (51.7)	0.55 (.395, .765)	.444(.257, .767)	−.190
Sex					
*Male*	355 (60.5)	231 (39.5)	1.0	1.0	
*Female*	19 (31.2)	42 (68.8)	0.294 (1.92, 5.98)	1.89(.683, 5.27)	.156
Age			1.02(.989, 1.01)	.990 (.970, 1.01)	−.002
Religion					
*Orthodox Christian*	372 (58.2)	266 (41.8)	1.0	1.0	
*Others*	2 (66.6)	1 (33.3)	1.43(.129, 15.8)	.610(.075, 4.96)	−.120
Marital status					
*Married*	339 (61.8)	210 (38.2)	1.0	1.0	
*Single*	10 (38.5)	16 (61.5)	.387(.172, .869)	1.71(.560, 5.22)	.116
*Divorced*	16 (39)	25 (61)	.396 (.207, .760)	1.11(.357, 3.48)	.025
*Widowed*	6 (25)	18 (75)	.206 (.081, .529)	.350(.085, 1.43)	−.256
Occupation					
*Farmers*	365 (58.5)	259 (41.5)	1.0	1.0	
*Others*	9 (40.9)	13 (59.1)	.491 (.207, 1.16)	.449(.175, 1.15)	−.196
Family size			1.245 (1.14, 1.35)	1.17 (1.02, 1.35)	.038
HH average health status					
*Poor*	76 (61.2)	48 (38.8)	1.0	1.0	
*Medium*	246 (57.4)	183 (42.6)	.849 (.564, 1.27)	.659 (.363, 1.19)	−.096
*Good*	57 (57.5)	42 (42.5)	.857 (.501, 1.46)	.380 (.179, .805)	−.235
chronic diseases					
*Do not exist*	105 (79.5)	27 (20.5)	1.0	1.0	
*Exist*	268 (53.3)	235 (46.7)	.293 (.186, .463)	3.42 (1.89, 6.19)	.252
Scheme benefit package adequacy					
*inadequate*	318 (58)	230 (42)	1.0	1.0	
*adequate*	57 (57.5)	42 (42.5)	.98 (.637, 1.51	2.17 (1.20,3.93)	.167
Institutional service quality					
*Poor*	241 (56.8)	183 (43.2)	1.0	1.0	
*Medium*	115 (61.5)	72 (38.5)	1.21 (.853, 1.72)	1.24 (.744, 2.07)	.050
*Good*	22 (55)	18 (45)	.928 (.484, 1.78)	1.84 (.709,4.78)	.131
Health service quality					
*Poor*	141 (60.8)	91 (39.2)	1.0	1.0	
*Medium*	189 (55.2)	153 (44.8)	0.797 (.568, 1.11)	3.20 (1.98, 5.15)	.269
*Good*	49 (62.8%)	29 (37.2)	1.09 (.642, 1.85)	3.69 (1.77, 7.69)	.255
CBHI awareness					
*Poor*	22 (81.5)	5 (18.5)	1.0	1.0	
*Medium*	30 (62.5)	18 (37.5)	0.37(.122, 1.17)	2.65(.735, 9.54)	.196
*Good*	327 (56.7)	250 (43.3)	0.29 (.111, .796)	4.90 (1.65, 14.4)	.376
HH social participation					
*Poor*	8 (57.1)	6 (42.9)	1.0	1.0	
*Medium*	85 (57.8)	62 (42.1)	1.02 (.340, 3.11)	.521(.093, 2.90)	−.157
*Good*	286 (58.3)	205 (41.7)	1.04 (.358, 3.06)	.748(.135, 4.13)	−.066
Social norm					
*Poor*	31 (59.6)	21 (40.4)	1.0	1.0	
*Medium*	173 (58)	125 (42)	.938 (.515, 1.70)	.868(.427, 1.76)	−.033
*Good*	172 (57.9)	125 (42.1)	.932(.512, 1.69)	1.02(.506, 2.09)	.006
Community solidarity					
*Poor*	75 (50.3)	74 (49.7)	1.0	1.0	
*Medium*	193 (63.9)	109 (36.1)	1.74 (1.17, 2.60)	3.02 (1.17, 2.60)	.252
*Good*	111 (55.2)	90 (44.8)	1.21 (.796, 1.86)	3.77(.796, 1.86)	.282
Community Trust					
*Poor*	4 (66.6)	2 (33.3)	1.0	1.0	
*Medium*	135 (58.2)	97 (41.8)	.696(.125, 3.87)	3.62(.634,20.7)	.280
*Good*	240 (57.9)	174 (42.1)	.690 (.125, 3.80)	5.65(.985, 32.4)	.401
Perceived living standard					
*Poor*	351 (58.8)	246 (41.2)	1.0	1.0	
*Rich*	27 (50)	27 (50)	.701(.401, 1.22)	1.15(.505, 2.63)	.033
HH wealth					
q*uintile1*(poorest)	58 (39.5)	89 (60.5)	1.0	1.0	
*quintile2 (poor)*	64 (58.2)	46 (41.8)	2.13 (1.29, 3.53)	2.05 (1.08, 3.91)	.156
*quintile3 (medium)*	78 (62.9)	46 (37.1)	2.60 (1.59, 4.25)	2.56 (1.36, 4.81)	.200
*quintile4(rich)*	75 (60.9)	48 (39.1)	2.39 (1.46, 3.91)	2.26 (1.17, 4.35)	.176
*quintile5 (very rich)*	95 (77.8)	27 (22.2)	5.39 (3.14, 9.27)	3.62 (1.67, 7.83)	.259

*** significant at 1% confidence level **significant at 5% confidence level * significant at less than 10% confidence level

In the study, scheme benefit package adequacy indicated a strong positive effect on enrollment. Household which perceived that the benefit package of the scheme was adequate were 2.17 times more likely to enroll compared with those who perceived inadequate benefit.

The results also revealed that respondents with good perception about the quality of health services were 3.69 times more likely to enroll in CBHI than respondents who perceived low health service quality (AOR = 3.69; CI = 1.77–7.69). Besides, households in the fifth quintile (the richest) were 3.62 times more likely to enroll than the poorest in the first quintile (AOR = 3.62; CI = 1.67–7.83).

In the social capital dimension solidarity was strongly associated with enrollment. Household who believed existence of good community solidarity were 3.77 times more likely to join than those who responded existence of weak community solidarity (AOR = 3.77; CI = 2.05–6.92).

## Discussion

The aim of this study was to assess the determinants of enrollment for community based health insurance in west Gojam Zone, Ethiopia. In this study, the enrollment rate was found to be 58.19%. Family size, health status of the family, scheme service adequacy, community solidarity, health institutions service quality, CBHI awareness, and wealth were significant determinants of households CBHI enrollment.

Household family size was a significant determinant of enrollment in the scheme. This finding is similar with those of other studies [7, 9, 11, 20, 23, 24].

Larger households were more likely to purchase the insurance than smaller ones. This was attributed to the huge financial burden that large households faced at times of risk. Besides, when many members family live together in a single unit, there may be the chance of sharing different ideas for making good decisions [25].

The result of this finding also revealed that household wealth as a determinant factor for enrolment in CBHI. This finding is in line with those of other studies [9, 11, 23, 26, 27]. As income (ablity to pay) is a critical factor in determining demand, the financial resource capacity of households and affordability of the premium are a first concern to enroll [28]. Thus, those who are in good socio-economic level would be in a better position to enroll than their counter parts.

Household scheme awareness on decision for enrollment was significant. This finding is consistent with other studies [7, 29–33]. Knowledge and understanding of insurance principles and the functioning of the CBHI facilitated both enrolment and renewal decisions. It is clear that if there is low literacy and lack of information related to CBHI among the community, there will be low enrolment and renewal decisions [7]. Thus, although the Government of Ethiopia has been conducting CBHI awareness creation activities through insurance agents in each district, the study indicate that as there is knowledge gap among the non-members of the scheme.

The variable on scheme benefit adequacy was also significantly associated with CBHI enrolment. This is similar to previous findings [7, 11, 30, 33]. This could be due to the direct benefits obtained from broadly defined benefit packages; as the scheme package increased, the clients benefit increased. The benefit package offered by CBHI schemes seemd to be globally appreciated by members. Including a new service in the health insurance scheme benefit package would positively influence enrolment [30].

In this study evidence of adverse selection was detected. The health status of household family members was negatively significant determinant of enrollment. This finding is consistent with previous findings [34, 35]. Households with more sick members were willing to enroll [24]. This could be due to the fact that people with poor health and chronic illnesses had greater perceived risk for care seeking and strong needs to join the scheme. However, this finding differed from that of a study conducted in Ethiopia [6] that has found that recent illnesses, incidence of chronic diseases, and self-assessed health status did not induce enrolment. This is perhaps a usual experience as voluntary insurance schemes are prone to adverse selection.

In this study, the quality of services of health institutions was a significant factor for enrollment decisions. This finding is in-line with other studies results [6, 33, 34]. This might be due to the direct benefits gained from the quality of services delivered by health institutions. That is because the primary objective of joining the scheme is to get high quality health service at affordable costs, quality of health care at public facilities such as patient reception (availability of health care providers, waiting time to be seen by a medical care provider, respect and consideration displayed by caregivers), prescriptions and availability of drugs and the rapidity of treatment results are strong determinant factors for membership [30].

Solidarity which encourages members who are susceptible to risk to pulltheir resources for common use is one of the key principles of a well functioning CBHI [15]. This study also indicated that community solidarity is a significant predictor of household enrollment decisions. This is in line with other studies [32, 36, 37]. The decision to join the scheme is high for individuals who perceive the existence of good community solidarity. That is, when there is a strong community solidarity, individuals value not only their own benefits but also that of community. Thus, building social solidarity in designing CBHI schemes will increase acceptability and uptake [24].

### Strength of the study

The study tried to comprehensively investigate the determinant factors for CBHI enrollment. Incorporating a social capital variable as a determinant factor is a particular strength of this study as it has been rarely done in the past. Moreover, the broad inclusion criteria used, the sampling technique (multi stage sampling), and the representativeness of the sample employed, we believe have confirmed the external validity of this study.

### Limitations of the study

Recall bias regarding the illness history of households in the past 3 months might be a limitation of the study. As a questionnaire survey, our work is subjected to information bias which is the intrinsic nature of the instrument. Moreover, it would have been also good if the study addressed the households health care needs.

## Conclusion

In this study, the CBHI enrollment rate of West Gojjam Zone was 58.19%. According to the regression results, family size, health status of a family, chronic disease in the household, scheme service adequacy, community solidarity, health institutions service quality, CBHI awareness, and wealth were significant determinants of households CBHI enrollment. Furthermore, the study indicated evidence of adverse selections. Therefore, in-depth and sustainable awareness creation programs on the scheme, stratified premium based on the economic status of households, incorporation of social capital factors, particularly community solidarity in the scheme implementation are vital to enhance sustainable enrollment.

Moreover, as family health status and chronic diseases were significant determinants of enrollment. The Government may need to look for options to make the scheme mandatory.

## Data Availability

Data that support the findings are available from the correspondence author on reasonable request.

## Abbreviations

AOR: Adjusted Odds Ratio
CBHI: Community Based Health Insurance
CI: Confidence Interval
COR: Crude Odds Ratio
OOP: Out of Pocket
UHC: Universal Health Coverage
